# Acetylene Acetogenin from *Porcelia macrocarpa* Exhibits Superior Efficacy against **Schistosoma mansoni* In Vivo*

**DOI:** 10.1021/acsomega.5c09262

**Published:** 2025-12-08

**Authors:** Dalete Christine S. Souza, Ivanildo A. Brito, Emerson A. Oliveira, Rayssa A. Cajás, Thainá R. Teixeira, Josué de Moraes, João Henrique G. Lago

**Affiliations:** † Federal University of ABC, Center for Natural and Human Sciences, Av. dos Estados, 5001, 09210-180 Santo André, SP, Brazil; ‡ Guarulhos University, Center for Research on Neglected Diseases, Praça Tereza Cristina, 88, 07023-070 Guarulhos, SP, Brazil; § Brasil University, Center for Research on Neglected Diseases, Rua Três de Dezembro, 38, 01012-010 São Paulo, SP, Brazil

## Abstract

Schistosomiasis affects nearly 250 million people, primarily in low-income regions. Aligned with the Sustainable Development Goals, the WHO roadmap highlights the urgent need for new therapeutic alternatives to praziquantel, the only available drug for schistosomiasis treatment. Natural products, including γ-lactones from Annonaceae species, have shown promising anthelmintic activity. The present work aimed to investigate the anthelmintic potential of the natural acetogenin 3-hydroxy-4-methylene-2-(*n-*eicos-11′-yn-19′-enyl)­but-2-enolide (HMB) isolated from *Porcelia macrocarpa* seeds, against *Schistosoma mansoni* . HMB was characterized by NMR and ESI-HRMS analysis, and its toxicity was assessed using mammalian cell lines and *Caenorhabditis elegans*. HMB exhibited schistosomicidal activity *in vitro* (EC_5__0_ = 19.3–24.6 μg/mL) with no detectable toxicity. *in vivo*, a single oral dose of 400 mg/kg resulted in a 92.9% reduction in worm burden, which exceeded the 88.2% reduction achieved with praziquantel. Additionally, a 94–100% reduction in egg burden was achieved. Remarkably, the efficacy of HMB was maintained at lower doses. For example, a 65% reduction in worm burden was achieved at 100 mg/kg, whereas praziquantel achieved only 15% at the same dose. Therefore, HMB from *P. macrocarpa* exhibits potent antischistosomal efficacy *in vivo*, supporting their further development as promising candidates for schistosomiasis therapy.

## Graphical abstract

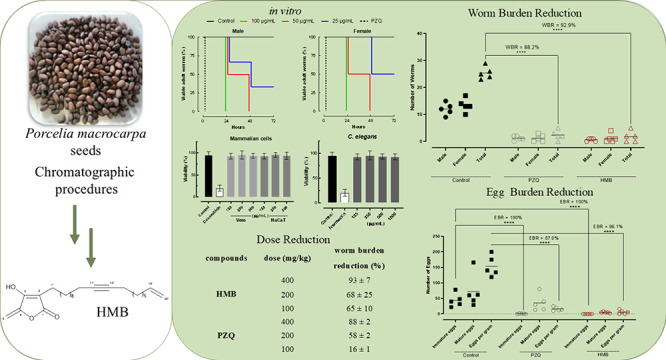

## 1 Introduction

Schistosomiasis, a debilitating parasitic disease caused by blood flukes of the genus *Schistosoma*, remains a major global public health concern, particularly in developing and/or underdeveloped countries where an estimated 800 million people are at risk of infection.^1^ In response to this persistent burden, the World Health Organization (WHO) launched a new roadmap for neglected tropical diseases (NTDs) in 2021, aiming to eliminate schistosomiasis as a public health problem by 2030, aligning with the Sustainable Development Goals.^2,3^ Key priorities in this roadmap include reducing the prevalence of high-intensity infections, improving diagnostic tools, and, critically, developing accessible therapeutic alternatives to praziquantel, the only currently available drug for schistosomiasis treatment.

*Schistosoma mansoni* is the etiological agent of hepato-intestinal schistosomiasis in Africa, the Middle East, the Caribbean, and the Americas. The morbidity associated with *S. mansoni* infection is influenced by several factors, including the intensity of the infection, the localization of the parasites, the number of eggs trapped in host tissues, and the immune response that ensues.^1^ Although administration with praziquantel has been a cornerstone of schistosomiasis control programs, relying exclusively on this single therapeutic agent is problematic due to emerging concerns about its reduced efficacy and the potential for resistance.^4^ These limitations underscore the urgent need for new anthelmintic agents to complement praziquantel in treatment protocols and help achieve sustainable disease control.^5^ Developing novel therapies for neglected tropical diseases (NTDs), such as schistosomiasis, faces significant challenges, including low commercial interest and high research and development costs.^6,7^ In this context, natural products have proven to be invaluable sources of structurally diverse and pharmacologically active compounds.^8,9^ Notably, approximately 60% of the antiparasitic agents approved between 1981 and 2019 were derived from natural sources. This fact highlights the ongoing importance of phytochemicals in the development of antiparasitic drugs.^10^

Recently, our group demonstrated that acetogenins from *Porcelia ponderosa* (Annonaceae) exhibited significant *in vivo* activity against *S. mansoni* , resulting in worm burden reductions (WBR) of 64.5%, 25.7%, and 12.4% at doses of 400, 200, and 100 mg/kg, respectively.^11^ In the present study, the natural acetogenin 3-hydroxy-4-methylene-2-(*n*-eicos-11′-yn-19′-enyl)­but-2-enolide (HMB) was isolated from the seeds of *P. macrocarpa*. HMB is structurally related to acetogenins previously identified in *P. ponderosa*, as these compounds share a γ-lactone core, though they differ in side-chain architecture. HMB has a C_20_ chain with a unique triple bond at C-11′ instead of a C_24_ chain with an enediyne system at C-13′. Given these structural features, as well as prior evidence of HMB antiparasitic activity against *Trypanosoma cruzi,* ^12–14^ and *Leishmania (L.) infantum*,^15,16^ the *in vitro* and *in vivo* efficacy of this acetogenin against *S. mansoni* was evaluated in a murine model of schistosomiasis.

## 2 Results and Discussion

### 2.1 Structural Identification of HMB

The seeds of *P. macrocarpa* yielded a pure acetogenin (99% by HPLC), identified through analysis of its NMR and ESI-HRMS data as 3-hydroxy-4-methylene-2-(*n*-eicos-11′-yn-19′-enyl)­but-2-enolide (HMB – Figure 1), as previously reported.^12^ HMB shares the characteristic γ-lactone pharmacophore of acetogenins from *P. ponderosa*,^11^ but it differs from these compounds due to its unique C_20_ side chain and alkyne functionality.

**Figure 1:**
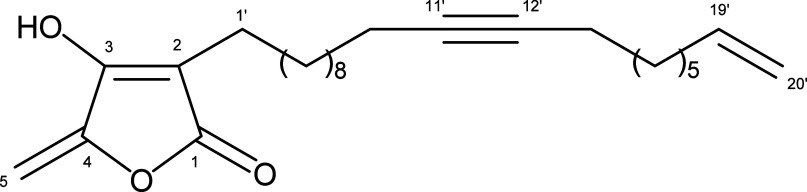
Structure of acetogenin HMB obtained from *P. macrocarpa* seeds.

### 2.2 HMB Impact on the Viability of Adult **S. mansoni* in vitro*

To evaluate schistosomicidal activity, adult *S. mansoni* worms were exposed to decreasing concentrations of HMB (Table 1 and Figure 2 – panel A). The positive control praziquantel caused rapid parasite death, while the DMSO-treated controls remained viable throughout the 72 h incubation period. Full mortality was observed at 100 μg/mL after 24 h and at 50 μg/mL after 48 h. At 25 μg/mL, partial killing was observed, with a nonsignificant trend toward higher sensitivity in males. The calculated EC_5__0_ values were 19.3 μg/mL for males and 24.6 μg/mL for females, with no statistically significant sex-dependent difference. These findings align with prior studies on other natural products, such as piplartine,^17^ licoflavone B,^18^ and epiisopilosine.^19^ These studies have demonstrated similar efficacy across both sexes.

**Figure 2:**
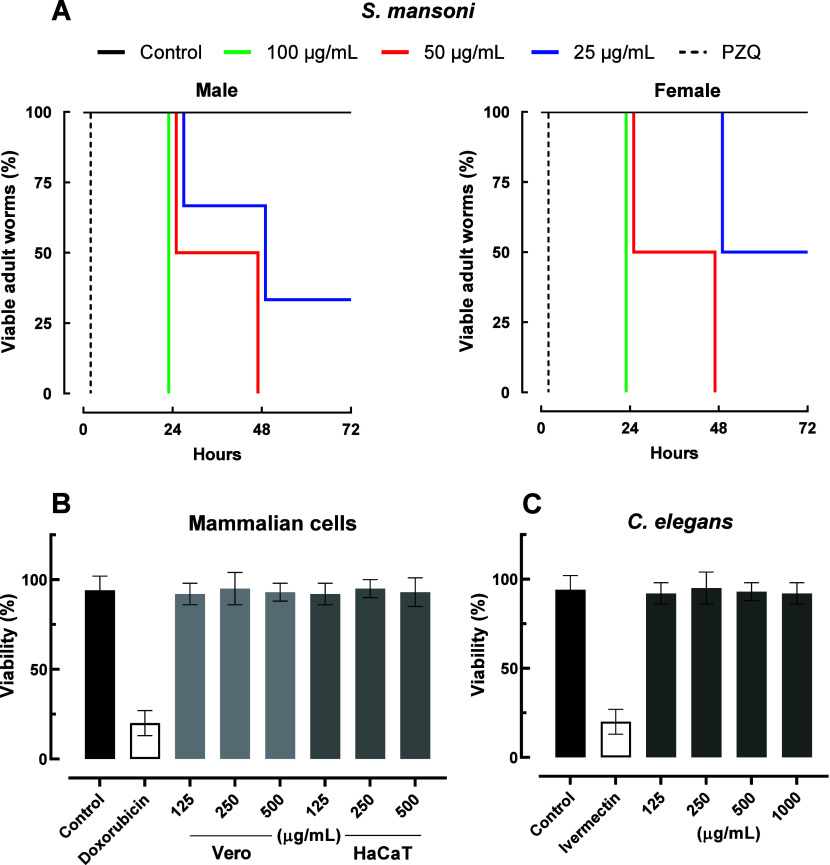
Viability of adult *Schistosoma mansoni* and toxicity profile of HMB. (A) Kaplan–Meier survival curves showing the viability of male and female adult worms over 72 h following exposure to the mixture. (B) *in vitro* cytotoxicity in Vero (monkey) and HaCaT (human) cell lines. (C) *in vivo* toxicity evaluation in*Caenorhabditis elegans* after 24 h of exposure. Doxorubicin (15 μg/mL) and ivermectin (5 μg/mL) were used as reference drugs. Controls were treated with 0.5% DMSO in RPMI-1640. Data represent mean ± SD of three independent experiments performed in triplicate.

**Table 1:** Antischistosomal Activity and Toxicity Profile of HMB Compared to Standard Reference Drugs^a^

	*S. mansoni* EC_50_ (μg/mL)		mammalian cells CC_50_ (μg/mL)		SI		
compounds	male	female	Vero	HaCaT	male	female	*C. elegans* LD_50_ (μg/mL)
HMB	19.3 ± 2.9	24.6 ± 2.6	>500	>500	>25.9	>20.3	>1000
praziquantel	0.9 ± 0.2	0.8 ± 0.3	>500	>500	>555.0	>625.5	ND
ivermectin	ND	ND	ND	ND	ND	ND	4.6 ± 1.1
doxorubicin	ND	ND	12.7 ± 2.8	8.9 ± 2.2	ND	ND	ND

a ND, not determined; Cytotoxicity in mammalian cells was assessed using Vero (animal origin) and HaCaT (human origin) cell lines; SI, selectivity index, calculated as the ratio of the CC_5__0_ value in Vero cells to the EC_5__0_ value against *S. mansoni* adult worms (SI = CC_5__0_/EC_5__0_). Values are expressed as mean ± standard deviation (S.D.) from at least three independent experiments performed in triplicate.

Despite being moderate, these EC_5__0_ values do not preclude strong *in vivo* efficacy. Many studies have shown that *in vitro* potency alone is not always a reliable predictor of *in vivo* outcomes due to the complexity of host metabolism and immune interactions, which can amplify or suppress the activity of tested compounds. Therefore, the next logical step was to evaluate the safety and efficacy of the system as a whole.^20,21^

### 2.3 HMB Displays Low Toxicity and High Selectivity

Safety profiling of HMB revealed no cytotoxicity toward Vero and HaCaT mammalian cell lines at concentrations up to 500 μg/mL, producing selectivity index (SI) values >20. Additionally, no toxic effects were detected in *C. elegans* even at the highest tested concentrations (Table 1 and Figure 2 – panel B). These findings are consistent with those observed for other bioactive natural products such as dihydrocitronellol,^22^ epiisopilosine^23^ and verrucosin,^24^ which have also demonstrated low cytotoxicity in both vertebrate and invertebrate models. Together, the lack of cytotoxicity in mammalian and nematode models strongly suggests that the tested HMB are safe and warrants further exploration as potential drugs.

### 2.4 Oral Administration of HMB Reduces Worm and Egg Burdens *in vivo*

Due to its favorable *in vitro* and toxicity profiles, HMB was evaluated *in vivo* next. A single oral dose of 400 mg/kg resulted in a remarkable 92.9% reduction in total worm burden (WBR), surpassing the 88.2% reduction produced by praziquantel under the same conditions (Figure 3). To our knowledge, this is the highest WBR reported for a natural product administered orally in a single dose at this concentration. Notably, this efficacy surpasses that of various natural products that have been tested under comparable conditions (a single oral dose of 400 mg/kg) such as spilanthol (42%),^25^ polygodial (44%),^26^ licarin A (50%),^27^ and 15β-senecioyl-oxy-*ent*-kaur-16-en-19-oic acid (62%).^28^ Despite sharing a γ-lactone scaffold with compounds from *P. ponderosa,* ^11^ the superior performance of HMB likely stems from its distinct side-chain structural features. These features include the presence of a unique triple bond and a shorter C_20_ aliphatic chain, which may modulate membrane interaction and metabolic stability.

**Figure 3:**
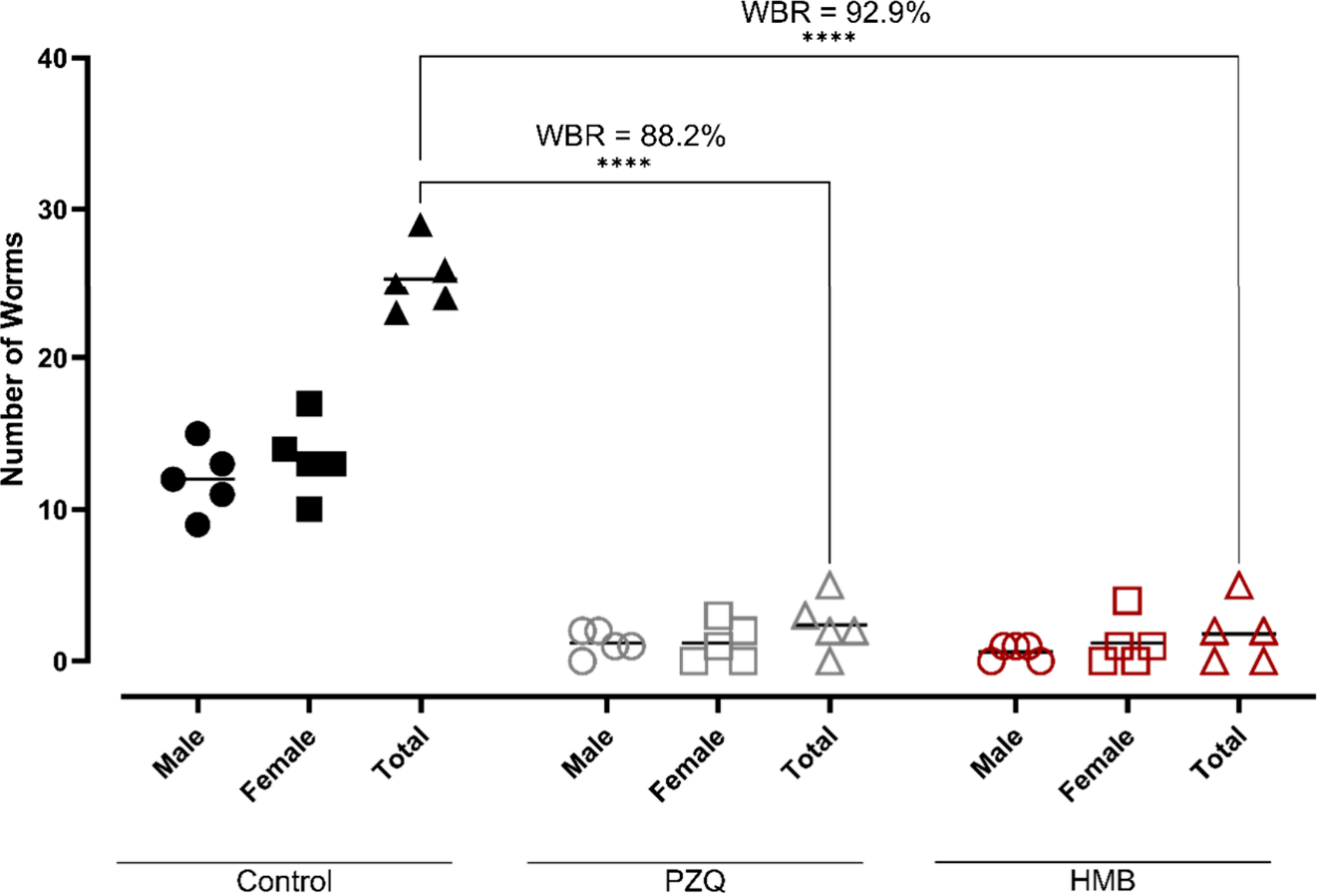
Effect of HMB on worm burden in mice infected with *Schistosoma mansoni* . Animals were treated orally with a single dose of the mixture (400 mg/kg) on day 49 postinfection. On day 63, worm burden reduction (WBR) was assessed by perfusion and stratified by sex (male and female). Each point represents an individual animal (*n* = 5 per group); horizontal bars indicate median values. Praziquantel (PZQ, 400 mg/kg) was used as a reference drug.

In addition to adult parasite clearance, egg burden reduction (EBR) was observed. HMB eliminated 100% of mature eggs in the intestinal wall, outperforming praziquantel (90.6%), and reduced immature eggs by nearly 100%, matching praziquantel performance (Figure 4). The fecal egg count decreased significantly, showing a 96.1% reduction compared to 87.0% with praziquantel (Figure 5). This comprehensive oviposition blockade is highly desirable in schistosomiasis treatment because it mitigates pathology and disrupts transmission.^29^

**Figure 4:**
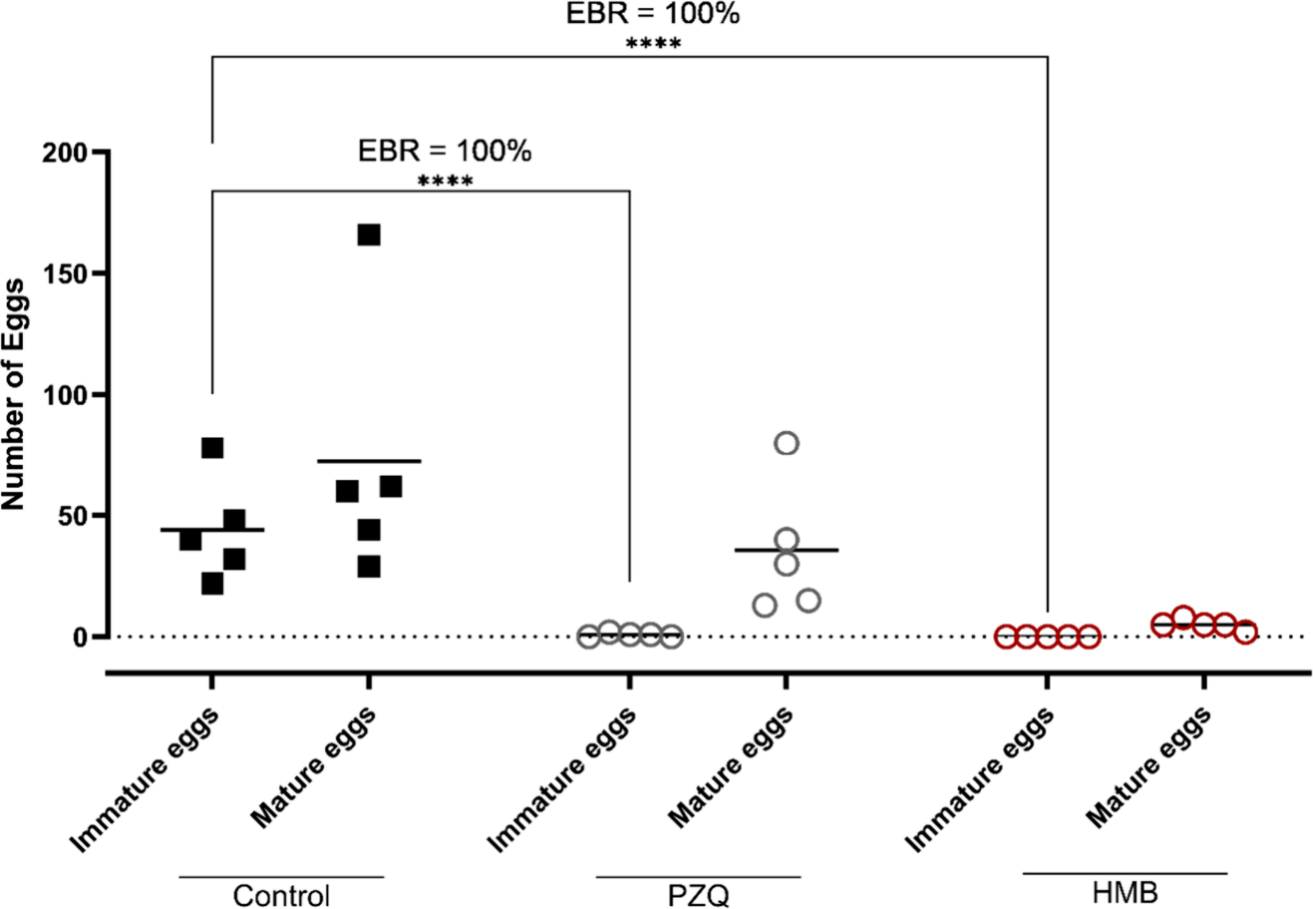
Effect of HMB on intestinal egg burden in mice infected with *Schistosoma mansoni* . Mice were treated orally with a single dose of the mixture (400 mg/kg) on day 49 postinfection. On day 63, egg burden reduction (EBR) was evaluated by quantifying immature and mature eggs in intestinal tissue. Each point represents an individual animal (*n* = 5 per group); horizontal bars indicate median values. Praziquantel (PZQ, 400 mg/kg) was used as a reference drug.

**Figure 5:**
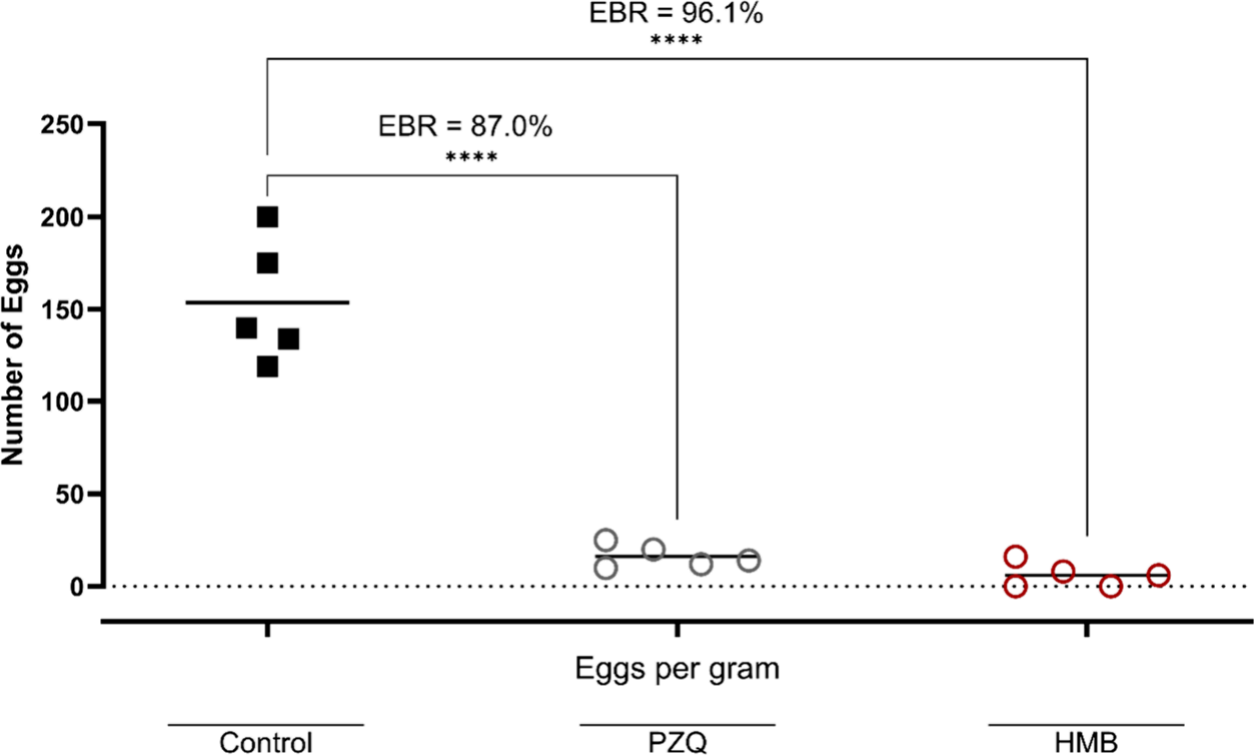
Effect of HMB on fecal egg burden in mice infected with *Schistosoma mansoni* . Mice were treated orally with a single dose of the mixture (400 mg/kg) on day 49 postinfection. On day 63, egg burden reduction (EBR) was assessed by quantifying eggs per gram (EPG) of feces. Each point represents an individual animal (*n* = 5 per group); horizontal bars indicate median values. Praziquantel (PZQ, 400 mg/kg) was used as a reference drug.

### 2.5 HMB Maintain Efficacy at Reduced Doses

To evaluate dose dependency, lower concentrations of HMB (200 and 100 mg/kg) were tested. At 200 mg/kg, the WBR was 68% whereas at 100 mg/kg, the WBR remained high at 65% (Table 2). In contrast, the efficacy of praziquantel decreased markedly at these doses (58% and 16%, respectively), as performed *P. ponderosa* acetogenins (25.7% and 12.4%, respectively).^11^ These results demonstrate the dose efficiency and robust anthelmintic activity of HMB, making it a promising candidate for further development as a low-dose, orally active agent against schistosomiasis.

**Table 2:** Effect of Different Doses of HMB and Positive Control Praziquantel on Worm Burdens in Mice Infected with *Schistosoma mansoni* ^a^

compounds	dose (mg/kg)	worm burden reduction (%)
HMB	400	93 ± 7
	200	68 ± 25
	100	65 ± 10
praziquantel	400	88 ± 2
	200	58 ± 2
	100	16 ± 1

a HMB was administered orally at three different doses (400, 200, and 100 mg/kg) on day 49 postinfection. On day 63, all animals were euthanized for analysis. Worm burden reduction was assessed by counting male and female worms.

The mechanism underlying the schistosomicidal effect of HMB has not yet been experimentally elucidated. However, evidence from related γ-lactone acetogenins provides valuable clues. Acetogenins isolated from *P. macrocarpa* were shown to disrupt the plasma-membrane electric potential and collapse the mitochondrial membrane potential in *T. cruzi*, leading to profound bioenergetic imbalance.^12^ Subsequent studies demonstrated that these compounds also alter cytosolic Ca^2^^+^ levels, increase reactive oxygen species (ROS) and ATP production, and disturb both plasma and mitochondrial membrane.^13^ Considering the structural similarity among *Porcelia* acetogenins, it is plausible that HMB induces parasite death through membrane depolarization, mitochondrial dysfunction, and ROS-mediated oxidative stress. Comparable results have been reported for other plant-derived compounds with antischistosomal activity. Extracts and isolated constituents from *Eremanhuts erythropappus* (Asteraceae) demonstrated potent *in vitro* and *in vivo* efficacy, achieving up to 86% reduction in worm burden without detectable cytotoxicity.^30^ Together, these findings reinforce the growing evidence that natural products constitute a valuable source of bioactive scaffolds for schistosomiasis chemotherapy.

## 3 Conclusions

This study is the first to demonstrate the potent antiparasitic activity of the natural acetogenin 3-hydroxy-4-methylene-2-(*n*-eicos-11′-yn-19′-enyl)­but-2-enolide (HMB), which was isolated from *P. macrocarpa* seeds. This γ-lactone-containing compound displays a potent ability to eliminate **S. mansoni*in vitro* and *in vivo*. It *in vivo* efficacy, characterized by a 92.9% reduction in worm burden and nearly complete suppression of egg output, exceeds that of praziquantel and most other natural products described in the literature. Importantly, its activity was retained at lower doses, highlighting its therapeutic efficiency. Toxicological assessments confirmed the safety of HMB in mammalian cells and the *C. elegans* model. Compared to previously studied acetogenins from *P. ponderosa*, structural differences in their aliphatic chains appear to enhance both efficacy and safety. Taken together, these findings suggest that HMB can be considered a promising prototype for treatment for schistosomiasis, aligning with the WHO 2030 roadmap goal of developing safe, effective, and accessible interventions for neglected tropical diseases. However, comprehensive evaluation of key parameters, including solubility, metabolic stability, and *in vivo* pharmacokinetic behavior, will be essential to guide formulation strategies and dosing optimization prior to any prospective clinical translation.

## 4 Materials and Methods

### 4.1 General

Column chromatography and analytical TLC were performed using silica gel 60 (63–210 mesh, Merck, Darmstadt, Germany) and silica gel F_254_ plates (Macherey-Nagel, Düren, Nordrhein-Westfalen, Germany), respectively. ^1^H and ^13^C NMR spectra were recorded on a Ultrashield Bruker Avance III spectrometer operating at 300 and 75 MHz, respectively. CDCl_3_ (Sigma-Aldrich) was used as the solvent and TMS was used as the internal standard. Chemical shifts are reported in δ (ppm) and coupling constants (*J*) in Hz. ESI-HRMS spectra were obtained using a Bruker Daltonics MicroTOF QII spectrometer. Reagents and solvents were used without further purification.

### 4.2 Plant Material, Extraction, and Isolation of HMB

Information concerning the botanical material and the procedure used for the isolation of HMB from *P. macrocarpa* seeds has been reported previously.^12^

### 4.3 Animals and Parasites

Three-week-old female Swiss mice were obtained from Anilab (São Paulo, Brazil). *Biomphalaria glabrata* snails and Swiss mice were used to maintain the life cycle of *Schistosoma mansoni* (BH strain) at the Guarulhos University (São Paulo, Brazil). The animals were kept under controlled conditions (25 °C, 50% humidity) and had ad libitum access to food and water.

### 4.4 *in vitro* Anthelmintic Assay

The *in vitro* anthelmintic activity of HMB was evaluated by determining the EC_50_ values, as previously reported.^25,31^

### 4.5 Cytotoxicity and *C. elegans* Toxicity Assays

The toxicity of HMB against Vero (African green monkey kidney) and HaCaT (human keratinocyte) cell lines was evaluated by determining CC_50_ values, as previously described. Selectivity index (SI) values were determined by dividing CC_50_ by EC_50_ values.^32^ Additionally, LD_5__0_ values obtained for *Caenorhabditis elegans* (strain N2) were calculated using a previously reported method.^31^

### 4.6 *in vivo* Antiparasitic Assay

The *in vivo* effect of HMB was evaluated as previously reported.^33^ Briefly, three-week-old female Swiss mice were subcutaneously infected with approximately 80 *S. mansoni* cercariae. On day 49 postinfection, the mice (n = 5 per group) received a single oral dose of 400 mg/kg of HMB in a EtOH:PBS 1:1 solution, while the control mice received the vehicle. On day 63, the animals were euthanized and the adult worms were recovered, sexed, and counted. Efficacy was also assessed by intestinal oogram analysis and fecal egg quantification. Additionally, the *in vivo* effects of HMB were evaluated at reduced doses of 200 and 100 mg/kg.

### 4.7 Randomization and Blinding

The mice were randomly assigned to groups and euthanized in a random order. Although treatment allocation was not blinded, data collection, including worm recovery and egg counts, was performed by different individuals. Two researchers not involved in experimental procedures conducted the data analysis independently.^34^

### 4.8 Statistical Analysis

The EC_5__0_, CC_5__0_, and LD_5__0_ values were calculated by plotting sigmoidal dose–response curves using GraphPad Prism 10.3.0. Kaplan–Meier survival analysis was applied to assess worm viability over time.^35^ *in vivo* results were analyzed using the nonparametric Kruskal–Wallis test, and differences were considered statistically significant at *P* < 0.05.^36^

### 4.9 Ethical Statement

All procedures were conducted in accordance with Brazilian legislation on animal experimentation (Law 11,790/2008) and the ARRIVE guidelines (NC3Rs). The protocol number 65/24 was approved by the Guarulhos University Ethics Committee on Animal Use (CEUA).
